# MWG-UNet++: Hybrid Transformer U-Net Model for Brain Tumor Segmentation in MRI Scans

**DOI:** 10.3390/bioengineering12020140

**Published:** 2025-01-31

**Authors:** Yu Lyu, Xiaolin Tian

**Affiliations:** School of Computer Science and Engineering, Faculty of Information Technology, Macau University of Science and Technology, Macao 999078, China; 2109853gii30002@student.must.edu.mo

**Keywords:** WGAN, brain tumor segmentation, MRI, U-Net, attention mechanism

## Abstract

The accurate segmentation of brain tumors from medical images is critical for diagnosis and treatment planning. However, traditional segmentation methods struggle with complex tumor shapes and inconsistent image quality which leads to suboptimal results. To address this challenge, we propose multiple tasking Wasserstein Generative Adversarial Network U-shape Network++ (MWG-UNet++) to brain tumor segmentation by integrating a U-Net architecture enhanced with transformer layers which combined with Wasserstein Generative Adversarial Networks (WGAN) for data augmentation. The proposed model called Residual Attention U-shaped Network (RAUNet) for brain tumor segmentation leverages the robust feature extraction capabilities of U-Net and the global context awareness provided by transformers to improve segmentation accuracy. Incorporating WGAN for data augmentation addresses the challenge of limited medical imaging datasets to generate high-quality synthetic images that enhance model training and generalization. Our comprehensive evaluation demonstrates that this hybrid model significantly improves segmentation performance. The RAUNet outperforms compared approaches by capturing long-range dependencies and considering spatial variations. The use of WGANs augments the dataset for resulting in robust training and improved resilience to overfitting. The average evaluation metric for brain tumor segmentation is 0.8965 which outperformed the compared methods.

## 1. Introduction

With the development of artificial intelligence, multiple deep learning methods propose for images processing with high speed and accuracy. Nowadays, artificial technology for medical image processing has became the most common method to improve the quality of medical images and speed of handling cases. Fusion of multiple image modalities improves the robustness and accuracy of segmentation by complementary information. Multiple types of medical images has been used for disease diagnosis, such as Radiography, Magnetic resonance imaging (MRI), Nuclear medicine, Elastography, Photoacoustic imaging, Tomography, Echocardiography, Functional near-infrared spectroscopy, Magnetic particle imaging and ultrasound. Different types of medical images focus on different types of disease diagnosis. The common method for abdominal organ detection is computer Tomography (CT) because of the comprehensive consideration of price and effect compared to other medical images. For the diagnosis of brain disease, MRI shows excellent potential with clear lesion imaging for normal tissue.

Deep learning methods for medical images segmentation automatically process large volumes of imaging data. In the early stage of medical images processing, manual segmentation for each case in the hospital which vary significantly between clinicians due to differences in experience and interpretation. Deep learning methods improve the efficiency of diagnosis with high-line suspicious parts in the medical images. The models of deep learning methods use extensive datasets for learning the corresponding of original images and segmentation results. Usually, we divide dataset for training to learn the pattern and testing to evaluate the accuracy and precision. Artificial intelligence has superior ability of reproducibility to detect patterns and features and identify subtle differences in tissue characteristics. The model of deep learning is continually improved and adapted as more data becomes available which allows them to learn from new cases and improve over time.

In recent years, deep learning has emerged as a powerful approach for medical image processing of both segmentation and classification. Convolutional Neural Networks (CNNs) is the original deep learning method for classification using convolution layers for image processing [1]. Due to the high capacity for feature extraction, CNN has been used for medical image segmentation and classification to improve quality and efficiency [2,3]. As the most powerful architecture for artificial intelligence, CNNs achieved a milestone compared to traditional methods for image processing. Automatic feature extraction improves efficiency to a large extent for massive data processing. CNNs have become foundational in medical image segmentation due to their ability to learn spatial hierarchies and capture intricate patterns in images. Due to the strong ability of shared weights and pooling layers, CNNs present talent performance in recognizing patterns. The high-level and low-level features are captured by different depths of the structure. In addition, CNNs achieved state-of-the-art results in various computer vision tasks. However, there are high computational costs and data requirements for the training model to avoid overfitting. In addition, CNNs primarily capture local patterns and fail to grasp global context without additional mechanisms. The fixed-size input images are required for standard CNNs architecture, which leads to loss of dominant information. Generative Adversarial Networks (GANs) [4] are also common channel for data augmentation as deep learning methods. They generate realistic synthetic images to mitigate the issue of limited annotated data. Ahmad et al. [5] proposed a new generative adversarial network for medical images from low resolution to super resolution. GAN-based synthetic medical image augmentation [6] presented a method for increased CNN performance in liver lesion classification.

To improve performance and efficiency, Fully Convolutional Networks (FCNs) [7] first replaced the convolution layer to be fully connected for pixel-by-pixel classification without fixed resolution. The elemental idea for end-to-end enable to streamline the pipeline by directly producing dense prediction output images with segmentation masks. The fully connected layers permit flexible size of input images without any modification to the architecture. FCNs leverage hierarchical features from multiple layers to capture both global and local features in the coding and decoding. Nevertheless, FCNs also has some drawback to overcome. Firstly, FCNs struggle with precise boundary delineation due to pooling operations. Secondly, large images processing at full resolution needs restore intermediate feature maps that cost high memory consumption. Lastly, the limited long-range context dependencies restrict for understanding of complex scenes for standard FCNs. The models pre-trained with large datasets and fine-tuning show brilliant performance for accuracy and efficiency. With the advancement of computing power, the architecture of deep learning has become comprehensive with a huge number of layers for extracting features to semantic segmentation. However, a large model for deep learning requires massive data to learn and train to avoid gradient explosion. The dataset of medical images possesses small amount of data with several patients from hospital due to privacy concerns, complexity of the annotation process and high price of capturing medical images.

In 2015, U-Net [8] proposed a delicate architecture for a small dataset with an encoder for the extraction of features and a decoder for feature fusion that uses a skip connection connected to shallow features and deep features to improve the performance of segmentation and classification. The brilliant idea of skip connection between encoder and decoder created a new path of splicing for shallow feature and deep feature. U-Net demonstrate a special structure of symmetric U-Shape for encode-and-decoder that warrant for capturing both contextual information and precise localization. Skip connection between corresponding layers in the encoder and decoder presents novel architecture to restore spatial information lost during pooling operation which improves performance of segmentation for boundaries. In medical images processing, accuracy is the crucial part of determination of performance for segmentation results. Skip connection is capable of combining deep features with high resolution to warrant high precision for fine structures. The variants of U-Net [9] have improved a lot for the segmentation and classification of medical images. U-Net ++ [10] formulated dense connection for short skip connection paths for adjacent convolution layers and upsampling convolutional blocks to extract features from different levels and scales to improve segmentation accuracy and reduce parameters. Among multiple area of image processing, the characterize of medical image is small data with different resolution because of divergent machine. U-Net is particularly effective with small datasets with data augmentation and shared wrights. Moreover, MZ Alom et al. [11] replaced convolutional layers with a recurrent convolutional layer for accumulation of features in recurrent networks. The nnUNet [12] is based on the U-Net architecture with sampling and normalization to remove the differentiation of different machines to ensure the stability of the building model. The flexibility across different domains of U-Net manifested in the ability of two-dimensional (2D) images processing and three-dimensional (3D) images processing. For volumetric data, U-Net is employed to leverage the spatial context across multiple slices. However, extensive use of skip connections and the dense feature maps generate high memory and computational demands. The U-Net, as improved architecture of FCNs, also struggle with limited long range context dependencies. 3D U-Net [13] extends the original 2D U-Net architecture for volumetric segmentation which utilizes 3D convolutions and sparse annotations to effectively capture spatial context dependencies.

For medical image processing, accuracy has played a significant role in comparing performance between different methods. The critical step in computer-aided diagnosis is accurate medical images segmentation for image-guided surgery, and treatment planning. In addition, the transformer [14] in the fields of medical images showed superiority in context for contextual semantic information. In the early stage of using CNN for image processing, segmentation and classification performance is restricted for computation and memory resources. Transformers improve performance in medical image segmentation by leveraging their ability to model long-range dependencies and capture global context through self-attention mechanisms. Transformers allow flexible input sizes of images for training data. What’s more, Transformers capture relationships between distant parts of an image for understanding complex information with subtle differences typical in medical images. The combination of deep learning methods with integrating domain knowledge could enhance interpretability and reliability. Dosovitskiy et al. [15] presented Vision Transformer (ViT) for combination of CNN and Transformer to upgrade in contextual semantic feature. ViT focus on images classification because of the superiority of spatial sematic feature to improve the ability of recognition. Correspondence Transformer for Matching Across Images (CoTr) [16] effectively connected convolution neural network and transformer for 3D medical images segmentation. CoTr extracted the feature from the CNN constructed and valid transformed decoder.

Therefore, the computation and spatial complexity have been degraded to process multiple scales and high-resolution medical images. Multiscale self-guided attention [17] proposed an attention mechanism for long-range context dependencies for fusion of local and global characteristics. The integration of transfers with CNNs demonstrate huge potential for retaining local feature extraction strengths of CNNs while adding the global attention benefits of transformers which leads to superior segmentation outcomes. The noise in medical images causes from old machine. The transformer focuses on relevant features across the entire image to reduce the influence of variations. The development for optimization of deep learning methods focus on real-time processing on edges deployment for pruning and quantization to improve efficiency for training and testing.

Transfuse [18] presented a combination of CNN and Transformer in parallel to effectively extract global dependencies and low spatial information and a new block called BiFusion to fuse multiple level feature. Gu et al. [19] introduced a new network with Convolutional attention-based Network (CA-Net) with Joint spatial attention module to promote performance in accuracy and interpretability. Incorporating attention mechanisms focus on relevant features to improve segmentation quality for complex anatomical regions. Deep learning for image processing need massive annotated data for training and testing to ensure stability of model. However, datasets of medical images always small compared with natural images because of morality and high cost of generating medical images. Transformers reduce the dependency on extensive labeled data by enhanced feature representation to use enormous unlabeled data for training and testing. Chen et al. [20] showed TransUnet for self attention block and global context information in vanilla U-Net to address the limitations of traditional Convolutional neural networks in modeling long-range dependencies and processing large-size images. However, CNN based model shows its limitation with long-term sematic information interaction due to fixed convolution computation problem. Therefore, Swin U-Net [21] proposed a transformer based U-Shaped architecture for 2D medical images segmentation which consist of encoder, decoder, bottleneck and skip connection. SelfReg-UNet [22] introduced Semantic Consistency Regularization (SCR) and Internal Feature Distillation (IFD) to reduce redundancy to improve the performance of medical images segmentation.

MRI is a common physical examination for brain tumor detection with excellent contrast resolution for identifying different types of soft tissues. The methods using deep learning automatically identify and delineate various structures within the brain to improve the efficiency of diagnosis. Mohammed et al. [23] discusses the use of hybrid techniques combining various features to enhance early diagnosis of brain tumors from MRI images. Ref. [24] explores the early diagnosis of brain tumors in MRI images by integrating deep learning and traditional machine learning techniques for improved detection accuracy. Ref. [25] evaluates the effectiveness of a hybrid technique on datasets for stroke and cerebral hemorrhage detection. The rapid detection of intracranial hemorrhages in CT images [26] also has excellent performance with deep learning. The early detection using MRI prevent the condition worsens further. Detector-based segmentation (DeSeg) [27] proposes a framework improving brain metastases(BMs) delineation in stereotactic radiosurgery which achieves high sensitivity and precision for small lesions and strong segmentation metrics for large ones efficiently. Rudie et al. [28] proposes 3D U-Net Convolutional Neural Network to detect intracranial metastases for stereotactic radiosurgery treatment planning. IGUANe [29] is a novel 3D model for harmonizing multi-center brain MR images demonstrating superior performance in age and Alzheimer’s disease-related tasks. Peng et al. [30] proposes 3D U-Net neural network to automatically segmentation for pediatric high-grade gliomas, medulloblastomas and other leptomeningeal seeding tumors.

Multiple medical images processing have been used for organ segmentation and classification for disease diagnosis and treatment planning. Our team is interested in brain tumor segmentation because brain tumors are a major challenge that threatens human health with complexity and diversity. Each type of brain tumor carries unique challenges for diagnosis and treatment. Therefore, the classification of brain tumors is not only an important cornerstone of medical images processing, but also the key for patients and their families to obtain accurate information and formulate reasonable treatment plans. The accurate segmentation of brain tumors from medical imaging is crucial for diagnosis and treatment. However, brain tumor segmentation approaches face significant hurdles due to the complex nature of tumor morphology and limited availability of annotated datasets. Traditional methods are not fully capture the intricate spatial features necessary for precise segmentation. To tackle these challenges, our study explores the integration of cutting-edge technologies in deep learning by combining U-Net architectures enhanced with transformer layers and Wasserstein Generative Adversarial Networks (WGANs). The hypothesis is that transformer layers significantly enhance the ability of model to understand spatial relationships within the data, while WGANs generate high-quality synthetic images to augment training datasets.

The major contributions of this paper are outlined as follows.

- The paper introduces a novel architecture called MWG-UNet++ that integrates transformers into the U-Net framework which called RAUNet for improving brain tumor segmentation by leveraging transformers’ ability to capture long-range dependencies and global context.
- By employing Wasserstein Generative Adversarial Networks (WGAN) for data augmentation, the study addresses the challenge of limited medical imaging datasets, generating high-quality synthetic images that enhance the training process and improve the model’s generalization capabilities.
- The combination of advanced segmentation techniques and effective data augmentation results in a model which is accurate and robust against anatomical variability in MRI scans.

## 2. Materials and Methods

In this section, we demonstrate the structure of our method for hybrid transformer U-Net model for brain tumor segmentation in MRI scans. The overall architecture of our method is illustrated in Section 2.1. The discriminator and generator of MWG-UNet++ is illustrated in Section 2.2. RAUNet is our main network for brain tumor segmentation in Section 2.3.

### 2.1. MWG-UNet++

Our team proposed MWG-UNet [31] for lung fields and heart segmentation with the Wasserstein generative adversarial network with CT images and outperformed a competition with other methods. In the early works, we focus on multiple scale of medical images segmentation for multiple organs segmentation. To improve the performance and the transfer ability for other medical image segmentation, we proposed an improved method called MWG-UNet++. We improved the original structure of MWG-UNet which replaced plain connection from encoder to decoder to DCA Cross-Attention (DCA) block to increase the efficiency of feature extraction. The details of the generator and discriminator will be demonstrated in the next parts of Section 2. The main architecture of MWG-UNet++ includes a generator and discriminator as usual GAN structure. The generator works for generating segmentation results in the stage of brain tumor segmentation and fake images which are highly similar to original medical images from training data. The discriminator also works to distinguish original images from fake images to obtain the parameters to improve the performance of brain tumor segmentation. The generator and discriminator corporation reveals the relationship of brain tumor and brain tissue to promote the accuracy of brain tumor segmentation. The brain cancer MRI is different from CT images which have four types of MRI for the same part to distinguish different tissue of the brain.

**Figure 1:**
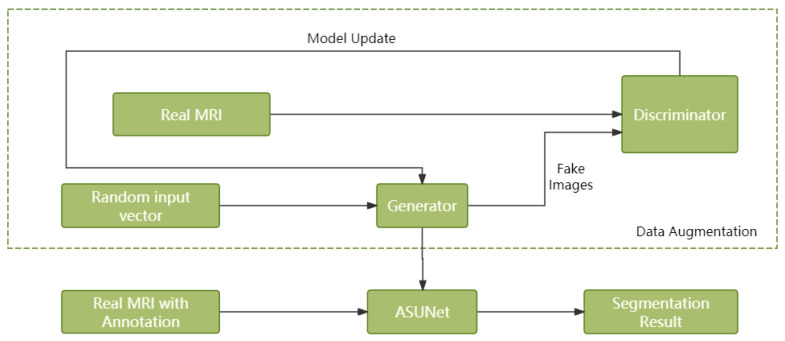
The flow chart for MWG-UNet++.

The Figure 1 is a flow chart for MWG-UNet++. The first step of our architecture is data augmentation with WGANs to ensure the next step of medical image segmentation. The original input of generator is random noise. The discriminator compared the difference between the output from generator and unlabeled original images to calculate Wasserstein distance and feed back related parameter to generator to improve the quality of images generation. The next step is using ASUNet for brain tumor segmentation with original labeled images and generated images. The detail of data augmentation and brain tumor segmentation is introduced in the next few sections.

### 2.2. The Discriminator and Generator of MWG-UNet++

The principle of GANs revolves around the concept of adversarial training involving two neural networks with a generator and a discriminator. These networks are set up in a game-theoretic scenario where their goals are opposing but interconnected.The generator’s objective is to create data that is indistinguishable from real data. It takes random input and transforms it through layers to produce synthetic outputs that mimic the distribution of real data. The discriminator acts as a classifier to differentiate between real data and fake data. The discriminator provides feedback to both itself and the generator based on its accuracy. The generator attempts to improve its ability to fool the discriminator during training. And the discriminator strives to better distinguish between real and generated data. This process is described as a minimum and maximum game with the following objective function:

(1) $$\min_{G}\max_{D}V\left(D,G\right)=E_{x∼P_{z}}\left[\log D\left(x\right)\right]+E_{z∼P_{z}}\left[\log\left(1-D\left(G\left(z\right)\right)\right)\right]$$

Here, *D*(*x*) represents the probability that input *x* is real. *G*(*z*) is the output of the generator given input noise *z*. The equilibrium occurs when the generator produces perfect fakes that are indistinguishable from real data. The principle of GANs provides fake medical images which highly similar to original medical images to enlarge dataset. At the core of a GANs introduced dual-network architecture comprising a generator and a discriminator. In the medical images processing, the biggest problem is limited images dataset with labeled due to the high cost and complexity. Deep learning methods need massive data for training model to avoid gradient explosion. GANs generate diverse and realistic synthetic images that expand these datasets to enhance the training of segmentation models. The generation of fake images improve images quality which reduces noise and artifacts to increase the accuracy of segmentation. Whereas, the gradient of training is unstable to mode collapse and gradient disappearance which failed for generation for insufficient diversity. WGANs are based on original GANs with Weight clipping to restraint weight to overcome the problem for unstable gradient. WGANs is based on GANs with restriction for weight which convert classification tasks into regression tasks. Wasserstein distance also called earth mover’s distance which measure the distance between different distribution. The purpose of WGANs for generator is trying to fit the Wasserstein distance between samples instead of generating fake images to trick discriminator. For discriminator of WGANs focus on shorten the Wasserstein distance between samples. The equation of Wasserstein distance is shown below:

(2) $$W\left(P_{\mathrm{data}\;},P_{G}\right)=\max_{D\in 1-Lipschitz}\left\{E_{x∼P_{\mathrm{data}\;}}\left[D\left(x\right)\right]-E_{x∼P_{G}}\left[D\left(x\right)\right]\right.$$

In original GANs, F-divergence is used for comparison between distribution. Wasserstein distance proposed a restriction that divergence will belong to 1−Lipschitz. The restriction of divergence provides continuity and differentiability of loss functions to improve stability of training model. Wasserstein distance proposed a meaningful gradient signal to decrease the probability of vanishing gradients and mode collapse. Further restriction for gradient is gradient penalty for generation data and original data to learn difference of distribution between generator and discriminator. The final improved Wasserstein distance is shown below:

(3) $$W\left(P_{\mathrm{data}\;},P_{G}\right)\approx \max_{D}\left\{E_{x∼P_{\mathrm{data}\;}}\left[D\left(x\right)\right]-E_{x∼P_{G}}\left[D\left(x\right)\right]\right.\left.-\lambda \int_{x}\max\left(0,∥\nabla_{x}D\left(x\right)∥-1\right)dx\right\}$$

The robustness of WGANs with gradient penalty showed outperformed performance than ever before. Weight clipping increase the difficulty of optimization in small dataset due to the restriction of weight. In the field of medical images segmentation, the complexity structure of organs need gradient penalty to learning the comprehensive distribution to ensure the accuracy for segmentation and classification. WGANs ensures diverse and comprehensive representations for data augmentation which is critical for medical images.

### 2.3. RAUNet

**Figure 2:**
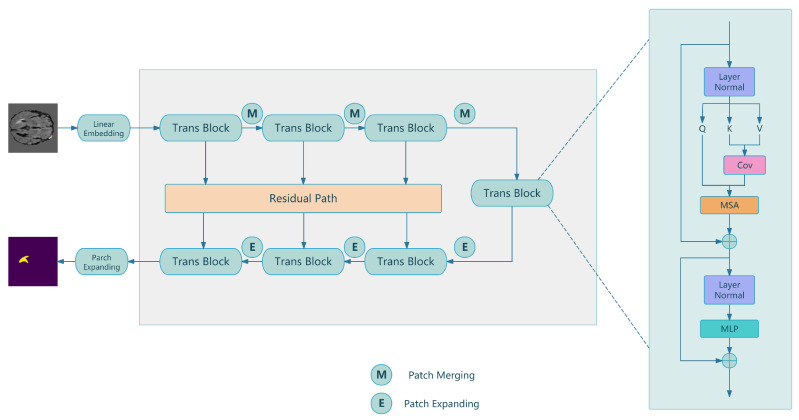
The architecture of RAUNet and the detail of Trans block.

In this papaer, we proposed a method call RAUNet for brain tumor segmentation which combined transformers and U-Net. The structure of our methods is shown in Figure 2. RAUNet is based on U-Net with encoder-to-decoder and residual path. The upper part with three trans block is encoder to extract hierarchical feature from the input images. The part below of Figure 2. is decoder of RAUNet to upsample feature which works for segmentation. Skip connection in the original U-Net has been changed to residual path to improve performance and efficiency of training network. Features from the encoder path are concatenated with the corresponding upsampled features from the decoder by residual path. It consists of several convolutional layers and batch normalization layers to learn the residual between input and output. The output of the residual path is added to the input of the main path to obtain the final output to enhance spatial information. For each trans block in RAUnet, we used several operations enhance global context understanding between consecutive layers by shifted window-based self-attention mechanisms. Embedding vector as input for trans block used *Q* (Query), *K* (Key) and *V* (Value) to calculate attention weight to update their value to find the relationship of related vector for segmentation. The Equation (4) introduced self attention mechanism as below:

(4) $$\mathrm{Attention}\left(Q,K,V\right)=\mathrm{softmax}\left(\frac{QK^{T}}{\sqrt{d_{k}}}\right)V$$

Here, the key of $d_{k}$ is 1. The goal of our task is brain tumor segmentation with high accuracy for position and size. The process of encoder is to extract feature to capture abstract representations. Each block uses layer normalization before the multi-head self-attention and the convolution layer components for stabilizing and accelerating training. These blocks contribute to a hierarchical representation for learning process and aggregating features from multiple scales. MSA (Multi-Head Self-Attention) is a core of trans block for attention mechanism to improve ability of extraction of spatial information to improve performance of brain tumor segmentation by multiple head self-attention. Attention is calculated independently using multiple attention heads. Each head learns different features of the input data. The residual connection of trans block ensured deep network to avoid to losing spatial dependencies. Each block processes tokens within fixed-size windows in parallel to reduce the computational complexity compared to full self-attention across the entire image. These blocks progressively reduce the spatial dimensions while increasing the feature channels and capturing contextual information effectively. The process reduces the spatial size but increases the depth of feature channels. The deepest layer acts as the bottleneck to reduce computational complexity and enhance feature extraction capability. For decoder of our architecture, the feature maps are upsampled using interpolation to recover spatial resolution step-by-step for brain organ segmentation. The final layer of output is a convolution layer to map high resolution feature to the desired number of output channels.

RAUNet combines transformers and U-Net with residual path which reduced computational from full self-attention network and increase ability at capturing long-range dependencies and contextual relationships. It merges the powerful hierarchy and multi-scale processing capabilities of U-Nets with the global modeling prowess of transformers and residual path with MRI images for brain tumor segmentation.

## 3. Results

This section illustrates our result of brain tumor segmentation with MWG-UNet++. The Section 3.1 presents the detail of dataset. The evaluation metrics is shown in Section 3.2. The Section 3.3 introduces final result of segmentation with table and figure.

### 3.1. Data Processing

We used Multimodal Brain Tumor Image Segmentation Benchmark (BraTS) [32] as 1251 cases for training and testing which is publicly available through the Medical Image Computing and Computer-Assisted Intervention Society (MICCAI) accessed via the official BraTS challenge website. The dataset specifically targets the segmentation of low and high grade gliomas into three critical sub-regions, which is the enhancing tumor, the whole tumor and the tumor’s surrounding edema. The BraTS includes a diverse collection of MRI scans gathered from multiple institutions worldwide to ensure a comprehensive representation of brain tumor heterogeneity. There three label for tumor in dataset. Enhancing Tumor (ET) indicates the parts of the tumor that enhance with contrast agent for active tumor growth. Tumor Core (TC) encompasses the enhancing tumor, non-enhancing solid tumor and necrotic tumor core without the surrounding edema. Whole Tumor(WT) includes all visible tumor tissues which combines the enhancing tumor, non-enhancing tumor, necrotic core and the peritumoral edema. The MRI in BraTS 2021 for brain tumor detection has four imaging sequences which consist of 1-Weighted Imaging (T1WI), T2-Weighted Imaging (T2WI), Fluid-Attenuated Inversion Recovery (FLAIR) and Diffusion-Weighted Imaging (DWI). T1WI offers high resolution anatomical detail to identify the structural boundaries of the brain and displacement caused by a tumor. The brain tumors take up the gadolinium contrast agent and appear brighter on T1-weighted images which helps in distinguishing tumor tissue from normal brain tissue and highlight areas of blood-brain barrier breakdown. T2-weighted images are particularly sensitive to changes in water content. Due to edema, the bright part is common in and around tumors which is easy to see tumor margins and surrounding tissue changes. Most tumors appear hyperintense on T2-weighted scans because of their higher water content compared to surrounding normal tissue. FLAIR imaging suppresses the bright signal of cerebrospinal fluid (CSF) which allows superior visualization of lesions adjacent to CSF-filled spaces for detecting cortical lesions and ventricles. DWI assesses the movement of water molecules within tissues. Tumors with high cellular density restrict water diffusion and appear hyperintense on DWI. In addition, DWI helps differentiate between types of tumors and other pathologies which is hard to detection with traditional MRI.

### 3.2. Evaluation Metrics

Evaluation metrics for medical images segmentation is comprehensive with multiple methods. We use Dice Similarity Coefficient for our evaluation of performance. The Dice Similarity Coefficient (DSC) is a metric used to quantify the similarity between two sets of data. The Dice Similarity Coefficient remains one of the most favored metrics for assessing segmentation quality due to its simplicity and effectiveness in capturing overlap. It has become a crucial measure in various fields such as computer vision in image segmentation tasks. The dataset is extensive to provide a robust basis for training and evaluating segmentation models. Mathematically, the Dice coefficient is defined as:

(5) $$\mathrm{DSC}=\frac{2\times |A\cap B|}{\left|A|+|B\right|}$$

where *A* is the set of predicted and *B* is the set of ground truth. In medical image analysis, the DSC is extensively used to evaluate the performance of segmentation algorithms. In this study, we use DSC to delineate anatomical structures in MRI scans which provides a quantitative assessment of how closely the algorithm’s output matches the ground truth annotations made by experts. In the realm of medical imaging, accurate segmentation is vital for diagnosis and monitoring disease progression. The DSC is favored because it balances sensitivity to both false positives and false negatives. It effectively captures true positive rates by equally weighting precision and recall. The DSC provides a reliable and intuitive means to compare and validate the fidelity of segmentation outputs.

### 3.3. Segmentation Result

In recent years, advances in medical imaging have significantly enhanced our ability to diagnose and treat various conditions, particularly in the field of oncology where accurate tumor detection and characterization are crucial. Table 1. presented showcases the comparative performance of several segmentation techniques. Each evaluated based on their ability to delineate different tumor regions: Enhancing Tumor (ET), Tumor Core (TC), and Whole Tumor (WT).

Accurate segmentation of these areas is vital for developing effective treatment plans. The E1D3 [33] demonstrates solid performance across all metrics which indicated its robustness in tumor segmentation tasks. However, it is surpassed by other methods in some specific evaluations. Fidon et al. [34] provides a noteworthy enhancement in WT segmentation which achieves the highest score among the methods compared for suggesting its potential usefulness in scenarios. Peiris et al. [35] offers competitive results, while slightly trailing in terms of average performance.

MVSI-Net [36] presents promising results with indicative of underlying technological strengths. VcaNet [37] displays exceptional accuracy in TC segmentation. Despite strong competition, the method developed by our team exhibits superior average performance. Our method demonstrates superior performance in the segmentation of tumors by its leading average score of 0.8965. It achieves the highest score in the Enhancing Tumor (ET) category with 0.8864 for indicating exceptional accuracy in identifying areas of active tumor growth. In Tumor Core (TC) and Whole Tumor (WT) metrics, our method scores 0.882 and 0.921 which showcases its reliable performance across different tumor regions. Compared to other methods, ours consistently ranks at or near the top across all evaluation criteria for highlighting its effectiveness and robustness in medical image segmentation tasks. This success is attributed to its integration of RAUNet for precise segmentation of brain tumor and WGANs for data augmentation. The remarkable ET score underscores its capacity to effectively identify enhancing tumors which are more challenging due to their dynamic and heterogeneous nature.

Each method’s distinct strengths suggest that future developments benefit from hybrid models that combine the best features of multiple approaches for fostering further enhancements in segmentation tasks. Furthermore, while our method currently leads in overall performance, the pursuit of comprehensive and clinically applicable solutions remains an ongoing challenge that will drive future research and development efforts in this field. Ultimately, the goal is to translate these technological advancements into tangible improvements in patient outcomes which ensures that every patient receives the most accurate diagnosis and the most effective treatment available.

**Figure 3:**
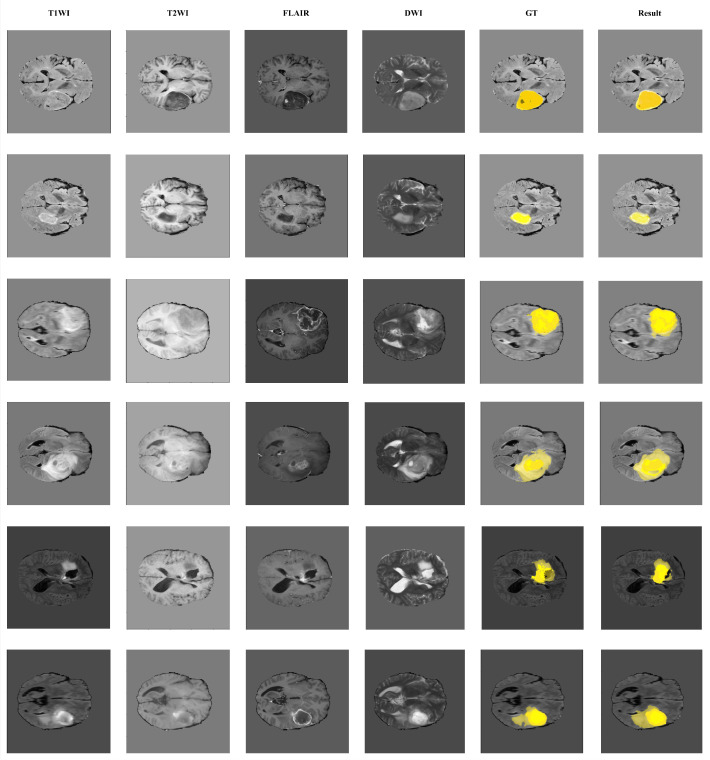
The brain tumor segmentation result with MWG-UNet++.

The Figure 3 is shown as the brain tumor segmentation result with MWG-UNet++ which presents a comprehensive visualization of various brain scans for demonstrating the effectiveness of the MWG-UNet++ in brain tumor segmentation. Each row corresponds to an individual patient case and includes a sequence of images from different MRI modalities: T1-weighted imaging (T1WI), T2-weighted imaging (T2WI), Fluid Attenuated Inversion Recovery (FLAIR), Diffusion Weighted Imaging (DWI), Ground Truth (GT), and the final segmentation results produced by the MWG-UNet++.

The first four columns display standard brain imaging techniques used in clinical settings. T1WI is shown first which provides clear anatomical details to help to distinguish between different types of tissue based on their intensity. The second column, T2WI, highlights fluid-filled spaces and lesions for offering a contrast that reveals edema. FLAIR presentes as the third image, suppresses cerebrospinal fluid signals for making it easier to detect lesions near ventricles. DWI displays in the fourth position which measures the diffusion of water molecules in tissue.

Figure 3 shows the brain tumor segmentation result with MWG-UNet++. The column T1WI is original brain MRI which prepares for automatic segmentation with MWG-UNet++. The column GT is ground truth for labeled brain tumor tissue. The column Result is our methods for brain tumor segmentation results. The comparison of other methods with brain MRI shows the improvement in many value of evaluation metric including the average value of result. However, the value of whole tumor is worse than Fidon et al. [34]. In the future work, we will experiment with different loss functions and improve training data quality with imaging processing to handle boundary ambiguity to improve performance for whole tumor with the surrounding edema.

The fifth column shows the Ground Truth (GT) which consists of manually annotated images. These annotations serve as a gold standard reference for evaluating the accuracy of the segmentation algorithm. Experts have delineated the regions of interest within the brain scans which identifies tumor boundaries and regions as the Enhancing Tumor (ET), Tumor Core (TC), and Whole Tumor (WT).

The final column displays the segmentation outcomes generated by MWG-UNet++. These results are visually overlaid on the brain scans with varying degrees of yellow indicating different tumor components. The most intense yellow highlights the Enhancing Tumor (ET) areas. A lighter shade represents the Tumor Core (TC) capturing both enhancing and non-enhancing solid tumor parts. The faintest yellow depicts the Whole Tumor (WT) encompassing all tumor-associated tissue, including edema and infiltrative regions.

This color-coding provides a quick visual assessment of segmentation capabilities across the three critical areas. Notably, the segmentation results align well with the GT annotations across different cases which underscores the precision and consistency. The accurate identification of these regions is paramount for effective diagnosis. By integrating information from multiple imaging modalities, MWG-UNet++ leverages the complementary strengths of each type. This multi-modal approach facilitates a more comprehensive understanding of complex tumor structures which enhances the ability to accurately delineate tumor boundaries.

### 3.4. Ablation

The Table 2 provides a performance comparison of RAUNet and RAUNet combined with WGANs for brain tumor segmentation using evaluation metrics for Enhancing Tumor (ET), Tumor Core (TC) and Whole Tumor (WT). The RAUNet achieves scores of 0.8632 for ET, 0.8905 for TC and 0.9076 for WT which results in an overall average score of 0.8871. In contrast, the RAUNet + WGAN shows an improved performance for ET at 0.8864 and WT at 0.921, while it slightly underperforms in the TC category with a score of 0.882. The integration of WGAN with RAUNet leads to a higher average score of 0.8965 which indicates that the addition of WGAN enhances the model’s capability in segmenting tumors more accurately across multiple metrics. This ablation study underscores the potential benefits of incorporating adversarial networks in improving the precision of medical imaging tasks by boosting ability to delineate complex tumor boundaries effectively.

## 4. Discussion

This paper discussed a novel methods called MWG-Unet++ for brain tumor segmentation with MRI scans and gained good performance compared with other methods. The main idea of our method is the combination of improved WGANs and U-Shaped Networks with transformers block which improved the skip connection from encoder to decoder. Our goal of using improved WGANs for data augmentation is to eliminate quantitative restriction of medical images. Massive data for training model avoid to vanishing gradients and mode collapse. Brain tumor segmentation works for computer auxiliary which is crucial for accuracy. Except for traditional data augmentation with translation and rotation, our team proposed deep learning methods for data augmentation. In order to ensure the quality of medical images generation, we used improved WGANs instead of traditional GANs.

We use RAUNet for brain tumor segmentation with brain MRI which gained improvement compared with other methods. The integration of transformer layers into the U-Net architecture for brain tumor segmentation represents a promising advancement in medical image analysis. This combination leverages the strengths of both convolutional neural networks and transformer models to enhance segmentation performance. The combination for U-Net and Transformers shows their ability to capture long-range dependencies and contextual information across the entire image. Traditional CNN-based U-Nets often focus on local features due to the limited receptive fields of convolutions which are inadequate for structures segmentation for whole tumors that exhibit substantial variability in size and shape. RAUNet mitigate this limitation with utilizing self-attention mechanisms which allows the model to integrate global context throughout the feature map. This comprehensive understanding of spatial relationships leads to more accurate delineation of tumor boundaries. The ability of RAUNet contributes to improved boundary delineation in complex tumor regions. Brain tumors possess irregular and indistinct borders which makes precise segmentation challenging. By embedding trans blocks within the U-Net, RAUNet leverages attention maps to emphasize critical areas around tumor peripheries.

Brain tumors present significant heterogeneity across patients for both in terms of appearance and anatomical location. MWG-UNet++ demonstrates robustness to variability which attributes to its dual approach of combining localized feature extraction with holistic spatial reasoning. This adaptability is crucial for generalizing across diverse datasets and improving reliability in clinical applications. Compared to conventional U-Nets or other CNN-driven architectures, MWG-UNet++ shows marked improvements in segmenting large-scale and intricate tumor regions. The inclusion of transformers addresses these gaps by providing a mechanism for integrating cross-channel and inter-pixel information.

The MWG-UNet++ merges the robust architecture of UNet++ with Wasserstein Generative Adversarial Networks (WGANs) to enhance medical image segmentation. Despite its promising advancements, several limitations need consideration which simultaneously highlight potential avenues for future research.

A primary limitation of MWG-UNet++ is its computational complexity. The combination of a complex segmentation network requires substantial computational resources and memory. This high demand restricts its use to institutions with access to advanced hardware and impede real-time processing capabilities essential. Future research look into optimizing algorithms and hardware acceleration to reduce computational load for accessible and efficient for broader applications.

Training stability remains another significant challenge. The instabilities WGANs occur during training phases. Research into adaptive learning rate schedules improved loss functions to enhance stability and performance. High-quality labeled data in medicine is scarce due to the expense and expertise required for annotation. Consequently, models trained on limited datasets may not generalize well across diverse clinical settings. To address this, future efforts focus on leveraging semi-supervised learning approaches to maximize efficiency from available data.

Interpretability is another critical factor in healthcare where understanding model decisions is crucial for clinician trust. The intricate structure of MWG-UNet++ complicates interpretability which limits its acceptance in medical practice. Developing methods for visualizing feature importance improves transparency. In the future work, integrating explainable AI (XAI) frameworks directly into the architecture help clinicians understand how the model processes input to reach specific conclusions.

Despite these challenges, MWG-UNet++ offers fertile ground for future research. Model simplification without sacrificing accuracy is a promising area. Model pruning will produce streamlined versions suitable for deployment in resource-limited environments. Transfer learning and domain adaptation will further extend the applicability to different medical imaging tasks and modalities to enhance versatility across various diagnostic scenarios.

Clinical validation is another vital step in transitioning from theoretical development to practical use. Collaborations with healthcare facilities for large-scale trials will provide insights into the effectiveness and reliability outside controlled research environments. These studies will drive model refinements that ensure it meets clinical needs and regulatory standards. In the future of our study, our method might have chance to evaluate in clinical validation to check whether it is valid.

In summary, MWG-UNet++ presents a compelling solution for brain tumor segmentation tasks which offers superior performance through sophisticated feature representation and robust handling of spatial information. While challenges regarding computational demands remain, the potential for improved diagnostic accuracy and personalized treatment planning makes this an exciting area of ongoing research and development.

## 5. Conclusions

In this work, we use improved WGANs for data augmentation at first step of image processing. RAUNet which is integration of transformers into the U-Net architecture for brain tumor segmentation represents a significant advancement in medical imaging analysis. This hybrid model combines the strengths of convolutional neural networks with the global attention mechanisms of transformers. By capturing long-range dependencies and integrating comprehensive contextual information, RAUNet addresses the challenges posed by the complex spatial variability inherent in brain tumors. Our experimental results demonstrate that this approach improves boundary delineation and robustness to anatomical variations which provides a more accurate and reliable tool for clinical applications. Despite the promising outcomes, MWG-UNet++ increases computational complexity which necessitates efficient implementation strategies to mitigate resource demands.

Future work focus on optimizing these processes and exploring lightweight models to enable broader deployment with limited computational infrastructure. We will aim to explore the integration of advanced transformer architectures to further enhance segmentation performance by capturing fine details and contextual information. Additionally, we plan to investigate semi-supervised learning to leverage unlabeled data to increase the model’s robustness and applicability in clinical settings.

Overall, MWG-UNet++ opens new avenues for research in medical image segmentation. It sets the stage for further innovations which enhances patient care through improved diagnostic accuracy and personalized treatment strategies. This study underscores the potential of advanced machine learning techniques to transform medical imaging practices.

## Figures and Tables

**Table 1:** The segmentation result for our method and compared methods.

Methods	Evaluation Metrics			Avg
	ET	TC	WT	
E1D3 [33]	0.822	0.865	0.924	0.8703
Fidon et al. [34]	0.84	0.84	0.927	0.879
Peiris et al. [35]	0.8139	0.8539	0.9077	0.8585
MVSI-Net [36]	0.817	0.875	0.876	0.856
VcaNet [37]	0.8325	0.8952	0.9203	0.8826
Our Method	0.8864	0.882	0.921	0.8965

**Table 2:** Performance comparison for ablation.

Methods	Evaluation Metrics			Avg
	ET	TC	WT	
RAUNet	0.8632	0.8905	0.9076	0.8871
RAUNet + WGANs	0.8864	0.882	0.921	0.8965

## Data Availability

The data were obtained from “Multi-Atlas Labeling Beyond the Cranial Vault—Workshop and Challenge”, available at https://www.synapse.org/#!Synapse:syn3193805/wiki/217789, accessed on 9 August 2022.

## References

[1] Shin H.C., Roth H.R., Gao M., Lu L., Xu Z., Nogues I., Summers R.M. (2016). Deep convolutional neural networks for computer-aided detection: CNN architectures, dataset characteristics and transfer learning. IEEE Trans. Med. Imaging.

[2] Zhao X., Wu Y., Song G., Li Z., Zhang Y., Fan Y. (2018). A deep learning model integrating FCNNs and CRFs for brain tumor segmentation. Med. Image Anal..

[3] Mehrtash A., Wells W.M., Tempany C.M., Abolmaesumi P., Kapur T. (2020). Confidence calibration and predictive uncertainty estimation for deep medical image segmentation. IEEE Trans. Med. Imaging.

[4] Kazeminia S., Baur C., Kuijper A., van Ginneken B., Navab N., Albarqouni S., Mukhopadhyay A. (2020). GANs for medical image analysis. Artif. Intell. Med..

[5] Ahmad W., Ali H., Shah Z., Azmat S. (2022). A new generative adversarial network for medical images super resolution. Sci. Rep..

[6] Frid-Adar M., Diamant I., Klang E., Amitai M., Goldberger J., Greenspan H. (2018). GAN-based synthetic medical image augmentation for increased CNN performance in liver lesion classification. Neurocomputing.

[7] Long J., Shelhamer E., Darrell T. Fully convolutional networks for semantic segmentation. Proceedings of the IEEE Conference on Computer Vision and Pattern Recognition.

[8] Ronneberger O., Fischer P., Brox T. U-net: Convolutional networks for biomedical image segmentation. Proceedings of the Medical Image Computing and Computer-Assisted Intervention—MICCAI 2015: 18th International Conference.

[9] Siddique N., Paheding S., Elkin C.P., Devabhaktuni V. (2021). U-net and its variants for medical image segmentation: A review of theory and applications. IEEE Access.

[10] Zhou Z., Rahman Siddiquee M.M., Tajbakhsh N., Liang J. Unet++: A nested u-net architecture for medical image segmentation. Proceedings of the Deep Learning in Medical Image Analysis and Multimodal Learning for Clinical Decision Support: 4th International Workshop, DLMIA 2018, and 8th International Workshop, ML-CDS 2018, Held in Conjunction with MICCAI 2018.

[11] Alom M.Z., Yakopcic C., Hasan M., Taha T.M., Asari V.K. (2019). Recurrent residual U-Net for medical image segmentation. J. Med. Imaging.

[12] Isensee F., Jaeger P.F., Kohl S.A., Petersen J., Maier-Hein K.H. (2021). nnU-Net: A self-configuring method for deep learning-based biomedical image segmentation. Nature methods.

[13] Çiçek Ö., Abdulkadir A., Lienkamp S.S., Brox T., Ronneberger O. 3D U-Net: Learning dense volumetric segmentation from sparse annotation. Proceedings of the Medical Image Computing and Computer-Assisted Intervention—MICCAI 2016: 19th International Conference.

[14] Shamshad F., Khan S., Zamir S.W., Khan M.H., Hayat M., Khan F.S., Fu H. (2023). Transformers in medical imaging: A survey. Med. Image Anal..

[15] Dosovitskiy A., Beyer L., Kolesnikov A., Weissenborn D., Zhai X., Unterthiner T., Dehghani M., Minderer M., Heigold G., Gelly S. An Image is Worth 16x16 Words: Transformers for Image Recognition at Scale. Proceedings of the International Conference on Learning Representations.

[16] Yuan F., Zhang Z., Fang Z. (2023). An effective CNN and Transformer complementary network for medical image segmentation. Pattern Recognit..

[17] Liu L., Wu F.X., Wang Y.P., Wang J. (2020). Multi-receptive-field CNN for semantic segmentation of medical images. IEEE J. Biomed. Health Inform..

[18] Zhang Y., Liu H., Hu Q. Transfuse: Fusing transformers and cnns for medical image segmentation. Proceedings of the Medical Image Computing and Computer Assisted Intervention—MICCAI 2021: 24th International Conference.

[19] Gu R., Wang G., Song T., Huang R., Aertsen M., Deprest J., Zhang S. (2020). CA-Net: Comprehensive attention convolutional neural networks for explainable medical image segmentation. IEEE Trans. Med. Imaging.

[20] Chen J., Mei J., Li X., Lu Y., Yu Q., Wei Q., Zhou Y. (2024). TransUNet: Rethinking the U-Net architecture design for medical image segmentation through the lens of transformers. Med. Image Anal..

[21] Cao H., Wang Y., Chen J., Jiang D., Zhang X., Tian Q., Wang M. Swin-unet: Unet-like pure transformer for medical image segmentation. Proceedings of the European Conference on Computer Vision.

[22] Zhu W., Chen X., Qiu P., Farazi M., Sotiras A., Razi A., Wang Y. SelfReg-UNet: Self-Regularized UNet for Medical Image Segmentation. Proceedings of the International Conference on Medical Image Computing and Computer-Assisted Intervention.

[23] Mohammed B.A., Senan E.M., Alshammari T.S., Alreshidi A., Alayba A.M., Alazmi M., Alsagri A.N. (2023). Hybrid techniques of analyzing mri images for early diagnosis of brain tumours based on hybrid features. Processes.

[24] Senan E.M., Jadhav M.E., Rassem T.H., Aljaloud A.S., Mohammed B.A., Al-Mekhlafi Z.G. (2022). Early diagnosis of brain tumour mri images using hybrid techniques between deep and machine learning. Comput. Math. Methods Med..

[25] Al-Mekhlafi Z.G., Senan E.M., Rassem T.H., Mohammed B.A., Makbol N.M., Alanazi A.A., Ghaleb F.A. (2022). Deep learning and machine learning for early detection of stroke and haemorrhage. Comput. Mater. Contin..

[26] Mohammed B.A., Senan E.M., Al-Mekhlafi Z.G., Rassem T.H., Makbol N.M., Alanazi A.A., Sallam A.A. (2022). Multi-method diagnosis of CT images for rapid detection of intracranial hemorrhages based on deep and hybrid learning. Electronics.

[27] Yu H., Zhang Z., Xia W., Liu Y., Liu L., Luo W., Zhang Y. (2023). DeSeg: Auto detector-based segmentation for brain metastases. Phys. Med. Biol..

[28] Rudie J.D., Weiss D.A., Colby J.B., Rauschecker A.M., Laguna B., Braunstein S., Villanueva-Meyer J.E. (2021). Three-dimensional U-Net convolutional neural network for detection and segmentation of intracranial metastases. Radiol. Artif. Intell..

[29] Roca V., Kuchcinski G., Pruvo J.P., Manouvriez D., Lopes R. (2025). IGUANe: A 3D generalizable CycleGAN for multicenter harmonization of brain MR images. Med. Image Anal..

[30] Peng J., Kim D.D., Patel J.B., Zeng X., Huang J., Chang K., Bai H.X. (2022). Deep learning-based automatic tumor burden assessment of pediatric high-grade gliomas, medulloblastomas, and other leptomeningeal seeding tumors. Neuro-Oncology.

[31] Lyu Y., Tian X. (2023). MWG-UNet: Hybrid Deep Learning Framework for Lung Fields and Heart Segmentation in Chest X-ray Images. Bioengineering.

[32] Baid U., Ghodasara S., Mohan S., Bilello M., Calabrese E., Colak E., Bakas S. (2021). The rsna-asnr-miccai brats 2021 benchmark on brain tumor segmentation and radiogenomic classification. arXiv.

[33] Bukhari S.T., Mohy-ud-Din H. E1D3 U-Net for Brain Tumor Segmentation: Submission to the RSNA-ASNR-MICCAI BraTS 2021 challenge. Proceedings of the Brainlesion: Glioma, Multiple Sclerosis, Stroke and Traumatic Brain Injuries: 7th International Workshop, BrainLes 2021.

[34] Fidon L., Shit S., Ezhov I., Paetzold J.C., Ourselin S., Vercauteren T. Generalized Wasserstein Dice Loss, Test-Time Augmentation, and Transformers for the BraTS 2021 Challenge. Proceedings of the Brainlesion: Glioma, Multiple Sclerosis, Stroke and Traumatic Brain Injuries: 7th International Workshop, BrainLes 2021.

[35] Peiris H., Chen Z., Egan G., Harandi M. Reciprocal Adversarial Learning for Brain Tumor Segmentation: A Solution to BraTS Challenge 2021 Segmentation Task. Proceedings of the Brainlesion: Glioma, Multiple Sclerosis, Stroke and Traumatic Brain Injuries: 7th International Workshop, BrainLes 2021.

[36] Sun J., Hu M., Wu X., Tang C., Lahza H., Wang S., Zhang Y. (2024). MVSI-Net: Multi-view attention and multi-scale feature interaction for brain tumor segmentation. Biomed. Signal Process. Control.

[37] Pan D., Shen J., Al-Huda Z., Al-Qaness M.A. (2025). VcaNet: Vision Transformer with fusion channel and spatial attention module for 3D brain tumor segmentation. Comput. Biol. Med..

